# Grating Theory Approach to Optics of Nanocomposites

**DOI:** 10.3390/ma14216359

**Published:** 2021-10-24

**Authors:** Subhajit Bej, Toni Saastamoinen, Yuri P. Svirko, Jari Turunen

**Affiliations:** 1 Photonics Laboratory, Physics Unit, Tampere University, FI-33720 Tampere, Finland; 2 Institute of Photonics, University of Eastern Finland, FI-80101 Joensuu, Finland; toni.saastamoinen@uef.fi (T.S.); yuri.svirko@uef.fi (Y.P.S.); jari.turunen@uef.fi (J.T.)

**Keywords:** nanocomposites, grating theory, Fourier Modal Method, novel nonlinear materials, deterministic aperiodic media, metasurface

## Abstract

Nanocomposites, i.e., materials comprising nano-sized entities embedded in a host matrix, can have tailored optical properties with applications in diverse fields such as photovoltaics, bio-sensing, and nonlinear optics. Effective medium approaches such as Maxwell-Garnett and Bruggemann theories, which are conventionally used for modeling the optical properties of nanocomposites, have limitations in terms of the shapes, volume fill fractions, sizes, and types of the nanoentities embedded in the host medium. We demonstrate that grating theory, in particular the Fourier Eigenmode Method, offers a viable alternative. The proposed technique based on grating theory presents nanocomposites as periodic structures composed of unit-cells containing a large and random collection of nanoentities. This approach allows us to include the effects of the finite wavelength of light and calculate the nanocomposite characteristics regardless of the morphology and volume fill fraction of the nano-inclusions. We demonstrate the performance of our approach by calculating the birefringence of porous silicon, linear absorption spectra of silver nanospheres arranged on a glass substrate, and nonlinear absorption spectra for a layer of silver nanorods embedded in a host polymer material having Kerr-type nonlinearity. The developed approach can also be applied to quasi-periodic structures with deterministic randomness or metasurfaces containing a large collection of elements with random arrangements inside their unit cells.

## 1. Introduction

Nanocomposites have recently emerged as viable alternatives to conventional bulk optical materials because they offer on-demand customization of linear and nonlinear optical properties by tailoring the size, shape, and types of nano-inclusions [1]. Among widely used nanocomposites are glassy materials embedding small metal and semiconductor nanoparticles [2,3,4,5], block copolymers [6], carbon composites with different preforms [7], carbon nanotube (CNT) reinforced nanomaterials [8], POSS (polyhedral oligomeric silsesquioxanes) nanoparticles in polymer matrix [9,10], and ceramics [11]. Diverse applications of these nanomaterials include photovoltaics [12], lasing [13], optical computing [14], data storage [15], gas sensing [16], printing [17], and hard coatings [18].

The optical response of nanocomposites can be customized by changing shapes [7] and mutual arrangements of the [19] nanoentities, i.e., by controlling Mie-type and plasmonic [20,21,22,23,24,25] resonances. These resonances significantly enhance the electromagnetic fields in the close vicinity of the nanoparticles and substantially alter the optical responses.

Although the advancement of fabrication techniques has made it feasible to prepare nanocomposites with customized properties [7,26,27], the consistent description of their optical properties is still lacking. This is partly because of an absence of the long-range order and strong coupling in the array of nanoentities comprising nanocomposites. To a large extent, the theory of nanocomposite optical response is still limited to the effective medium models (e.g., Maxwell Garnett (MG) model [28]), which are based on quasi-static approximation and can be employed only at low volume fractions of the inclusions [29]. Moreover, MG theory fails to describe the dependence of the plasmon resonance on the concentration and size of the nanoparticles. In the vicinity of the percolation threshold, when the large clusters of nanoparticles and their long-range coupling essentially contribute to the optical response, MG theory cannot provide a consistent picture of the light-matter interaction [30]. Similarly, Bruggeman theory [31], which is conventionally used for a high concentration of nanoparticles or nanovoids, also fails to predict the positions of the optical resonances and their dependence on the concentration and properties of the nanoinclusions. As a result, nowadays, calculating the linear and nonlinear refractive index and absorption coefficient of nanocomposites often relies on numerical simulations [32,33], including machine learning-assisted approaches, which require an enormous amount of experimental training datasets [34,35,36] and are hardly feasible. This makes it essential to develop theoretical models and/or numerical techniques capable of predicting the optical properties of nanocomposites and of bridging the gap between optical design and material fabrication.

This article proposes an approach for modeling the linear and nonlinear optical properties of nanocomposites by exploring the achievements of the grating theory. Specifically, we propose to employ the Fourier Modal Method (FMM), which provides rigorous solutions of diffraction problems for structures with two-dimensional transverse periodicity [37,38]. For applying FMM to media with random permittivity variations, we introduce a large unit cell containing a representative collection of nanoinclusions. By averaging over ensembles of such unit cells, we can determine the optical properties of the nanocomposite. With a sufficiently large unit cell, results obtained for a single realization are good approximations of the ensemble average. This method can also produce a large number of accurate training datasets for different unit cell morphologies for machine learning-assisted optimization approaches. Obtaining a similar number of training datasets from optical measurements would be extremely challenging. Furthermore, our modeling approach is well suited to study the optical properties of deterministic aperiodic media (DANS) with unusual spectral features. With the aid of this software, the spectral response of DANS media can be interpolated in a tunable fashion between perfectly periodic crystals and disordered random media [39] and hence will provide novel opportunities to explore and manipulate light-matter interactions at the nanoscale. Photonic metasurfaces with a large collection of elements inside their unit cell can also be accurately designed following our approach.

We introduce the modeling approach in Section 2 and subsequently apply it to model linear and nonlinear optical properties of several types of nanocomposites. First, we numerically demonstrate the dependence of birefringence in porous silicon (por-Si) on the size, shape, density, and spatial arrangement of the nanovoids. We then visualize hot spots in glass-metal nanocomposites (GMN) containing metal nanospheres on a glass substrate and compute the ensuing absorption spectra. As a final example, we estimate the effective Kerr nonlinearity of a nanocomposite consisting of silver nanorods in a nonlinear dielectric host medium by studying the change in absorption spectra with an increase of the incoming light field intensity.

## 2. Methodology

**Figure 1:**
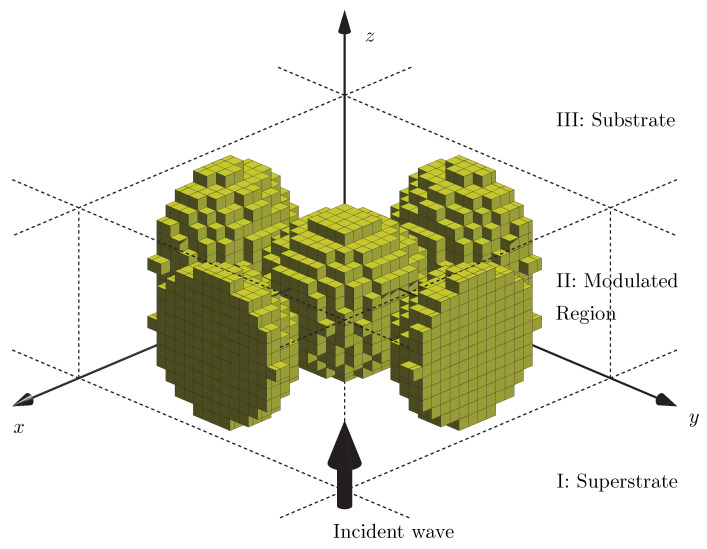
An illustration of the rigorous numerical modeling of nanostructures, where the 3D space is divided into (I) superstrate, (II) modulated region and (III) substrate. The permittivity-modulated structure in region II is quantized in cuboid blocks, each with a constant value of the dielectric permittivity.

In rigorous numerical solution of grating diffraction problems, the 3D space is divided into three regions as illustrated in Figure 1: the superstrate I, which is a transparent semi-infinite half-space containing the incident field, the index-modulated region II, and the (possibly opaque) substrate III. The solution generally involves writing separate expressions for fields in all three regions and then applying electromagnetic boundary conditions at the interfaces. This procedure typically leads to a set of linear algebraic equations, which can be solved to retrieve the electromagnetic field inside region II, as well as the diffracted fields in the substrate and the superstrate.

In modal methods, the field in region II is represented in terms of self-sustainable pseudo-periodic modes, which individually satisfy Maxwell’s equations and obey the internal boundary conditions. The complete solution is their superposition, with modal weights determined by the external electromagnetic boundary conditions at interfaces I–II and II–III. In FMM [37,38], region II is divided into a finite number of sub-regions in the nominal propagation direction (*z*) of light, in each of which the permittivity is assumed to be *z*-invariant and its transverse (xy) distribution is expanded into a Fourier series. Hence, the eigenmodes in each sub-region can be expressed in the form of pseudo-periodic Floquet-Fourier series. For monochromatic plane wave incidence, any modal field component $U_{i}$ (i=1,2,3) inside a sub-region can be mathematically expressed in the following form

(1) $$\begin{array}{cc} U_{i}\left(x,y,z\right) & =\sum_{pqm}v_{m}U_{i,mpq}\exp\left[i\left(k_{1,p}x+k_{2,q}y+\gamma_{m}z\right)\right], \end{array}$$

where, $v_{m}$ is the complex amplitude of the *m*-th mode, $\gamma_{m}$ is the propagation constant associated with the the *m*-th mode, $U_{i,mpq}$ is the transverse distribution of the mode *m* and

(2) $$\begin{array}{cccc} k_{1,p} & =k_{1,0}+p2\pi /d_{1}, & k_{2,q} & =k_{2,0}+q2\pi /d_{2}. \end{array}$$

$k_{1,0}$ and $k_{2,0}$ are the components of the incident wave vector along *x* and *y* axes respectively, p,q=0,±1,±2,…, and $d_{j}$ (j=1,2) are the grating periodicities along *x* and *y* directions respectively. Whereas fields in regions I and III are described by the usual Rayleigh (plane-wave) expansions. Use of the recursive S-matrix propagation algorithm [40,41] provides a numerically stable solution of the boundary value problems at all interfaces. A detailed mathematical formulation of FMM is out of the scope of this article and can be found in references [37,40,42,43].

**Figure 2:**
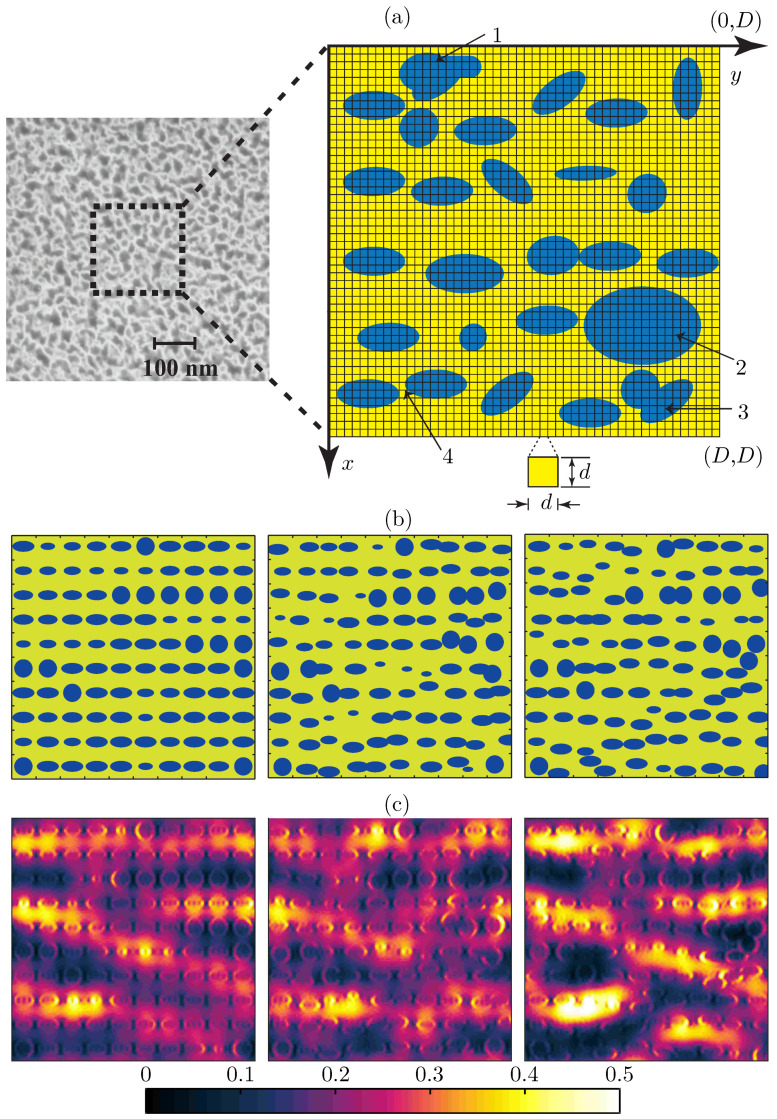
Modeling a porous silicon layer with rigorous grating theory—(**a**) (**Left**): Scanning electron micrograph showing a top-view of the porous-silicon layer, (**Right**): An illustration of the numerical building block (unit-cell) of the porous silicon monolayer. As illustrated, the unit-cell of size D×D (the area inside the black dotted square in the micrograph) is divided into small square grids, each of size d×d. Dielectric permittivity inside each smaller grid is assumed to be constant. In the proposed numerical approach, one can take the dimensions *d* of these square grids to be infinitesimal to model smoothly varying material boundaries accurately. As illustrated, the material building block can model air voids with irregular geometries (1), large air pores (2), overlapped air pores (3), and two neighboring air voids with ultra-small interspacing (4). (**b**) Three distinct/deterministically random pore arrangements inside one unit-cell of size D×D, with the same fill fractions of air voids. (**c**) *E*-field intensity maps immediately behind the porous layer (in silicon substrate), normalized to the incident *E*-field intensity. The plots correspond to the unit-cell configurations in (**b**), each with a cell size of D×D=2 μm×2 μm. An incident wave with λ=1.55 μm and *E*-field polarized along *y* is assumed.

To model a nanocomposite with FMM, we create a unit cell in the transverse xy plane as a building block for the whole composite medium and slice region II into thin layers in the *z* direction, as illustrated in Figure 1 and Figure 2 (right). The size of the unit cell is D×D, where *D* is comparable to or larger than the wavelength of light (λ), and it is divided (transversely) into square-shaped sub-cells of size d×d, where d≪λ. The refractive index in each sub-cell is assumed to possess a constant value, and by choosing a sufficiently small value of *d* we can model the boundary of a particle of arbitrary transverse shape at any desired precision. This approach naturally allows us to consider random arrangements of particles inside the unit cell, including long-range clustering of nanoparticles. There is no upper/lower bound on the size of individual nanoparticles, unless they are so small that quantum effects come into play. Furthermore, inclusions and/or host materials with Kerr-type optical nonlinearity can be rigorously modeled following the approach in reference [43].

From computational point of view, each layer along the *z*-direction must be thin enough, and the transverse (xy plane) sampling must be fine enough to ensure numerical convergence. In FMM, the computational workload increases linearly with the number of slices in the *z* direction. However, the fineness of transverse meshing does not affect the workload significantly (see Appendix A and Appendix B for further computational details/numerical convergence studies).

## 3. Numerical Experiments

We proceed to apply the method introduced in Section 2 for studying three different types of composite materials: (I) por-Si nanocomposites with customized unit-cells, (II) Ag nanospheres arranged on a glass substrate with diffrent arrangements of the nanospheres inside the unit-cells, and (III) periodic silver nanorods embedded inside a Kerr-type nonlinear polymer host matrix.

### 3.1. Porous Silicon

Por-Si nanocomposites contain nanovoids of different sizes and shapes, prepared by electrochemical or chemical etching of crystalline silicon substrates [44,45,46]. Depending on the pore dimensions (*r*), por-Si can be classified into three categories: microporous (r<5 nm), mesoporous (r≈ 5–100 nm), and macroporous (r>100 nm). A scanning electron micrograph (top view) of a monolayer mesoporous silicon nanocomposite is shown in Figure 2a (left). In what follows, we assume that the minimum pore dimension is at least a few nanometers, i.e., quantum effects do not play any role in the optical response of the nanocomposite. Effective-medium theories can be applied to accurately estimate the optical properties of por-Si only in the quasi-static limit i.e., for r≪λ, but finite wavelength effects come into play for mesoporous and macroporous samples i.e., when the size of the pores become comparable to the wavelength of excitation (r∼λ). Moreover, there is lack of a single effective-medium theory which can be applied for samples with very low (a few percent) to very high (≥50%) filling of air pores [25].

As a case study, we first take an example of a microporous silicon substrate with r<5 nm. To mimic the structure, we consider unit cells of size 200 nm×200 nm (D=200 nm) with three distinct internal structures/arrangements of the air pores as shown in Figure 2b. The air pores are assumed to have cylindrical profiles and only a single layer of pores is considered. The overall fractional volume (fill factor) of pores, in either case, is assumed to be the same, and using three different cell configurations, we study the effects of random pore arrangements only on the birefringence properties of porous Si monolayers. The layer thickness is 30 nm. The maximum pore size in all three cases is 18 nm and the refractive index of silicon is taken to be $n_{\mathrm{Si}}=3.5$. We assume normally incident plane-wave illumination at λ=1550 nm ensuring the validity of the quasi-static limit i.e., λ≫r. FMM simulations confirm that the microporous layers do not diffract light into higher orders as D<λ and hence can be modeled by the effective medium approach, which treats the porous layers as optically anisotropic homogeneous effective media. The effective indices of these composites for *y*- and *x*-polarized incident fields can be determined by treating region II as a single-layer thin film. The calculated effective indices of all three configurations in Figure 2b for *y*-polarized illumination are the same ($N_{y}=2.31$). However, for *x*-polarized illumination, the effective indices corresponding to the configurations in Figure 2b (left, middle, and right) are 2.25, 2.29, and 2.26 respectively. Hence the birefringence characterized by the difference $N_{y}-N_{x}$ depends on the chosen configuration even though the volume fractions of air pores are the same in all three configurations. Hence we see that the effective medium theories do not reveal complete information about the porous layers even in the quasi-static limit. The error grows linearly with the layer thickness.

Next, we study a mesoporous silicon monolayer with unit cell structures identical to those in Figure 2b, but with all feature sizes 10 times larger (cell size D=2000 nm, largest air pore dimension 180 nm, layer thickness 300 nm) than the previously studied example. Simulations with plane wave incidence (λ=1550 nm) show that the mesoporous composites can not be correctly described in terms of effective indices as the porous layers diffract light into higher orders of the grating defined by the unit cell (D>λ). The sum of the diffraction efficiencies in direct (0-th order) reflection and transmission lies in the range 92–96% and the rest (4–8%) is diffracted into non-zero orders. The birefringence in direct transmission, measured as the phase difference $\phi_{x00}-\phi_{y00}$ of the 0-th transmitted order between *x* and *y* polarized plane wave illumination, is 0.43, 0.3, and 0.35 radians for the three different configurations (left, middle, and right) respectively. As porous silicon nanocomposite is a mixture of a high index (Si, n=3.5) and a low index material (air, n=1), localized electric fields inside the porous layer, which become prominent especially for mesoporous and macroporous structures, strongly modify the optical properties of these nanostructured materials. These localized electric field effects are manifested in the form of hot spots, i.e., regions with anomalously high electric field strengths. Figure 2c visualizes such hot spots by illustrating distributions of the total electric energy density of the transmitted field for configurations in Figure 2b. Using effective medium approaches, it is not possible to study such distributions of hot spots.

### 3.2. Silver (Ag) Nanospheres Arranged on a Glass Substrate

Next, we study a planar (monolayer) GMN composed of ensembles of spherical silver nanoparticles in a dielectric host medium. Intra-particle plasmon resonances for such nanomaterials occur around 400 nm, and the absorption spectra (at resonance) depend strongly on the surrounding dielectric medium, the morphology of the Ag nanoparticles, and the metal fill fraction. If the radius *a* of the spherical nanoparticles is much smaller than the wavelength i.e., a≪λ and the interparticle distance *c* satisfy the conditions a≪c≪λ, the effective dielectric constant $\varepsilon_{\mathrm{eff}}$ of the bulk nanocomposite follows the well-known Maxwell Garnett (MG) theory:

(3) $$\frac{\varepsilon_{\mathrm{eff}}-\varepsilon_{h}}{\varepsilon_{\mathrm{eff}}+2\varepsilon_{h}}=f_{i}\frac{\varepsilon_{i}-\varepsilon_{h}}{\varepsilon_{i}+2\varepsilon_{h}},$$

where $\varepsilon_{h}$ and $\varepsilon_{i}$ are the dielectric constants of the host and the inclusions, respectively, and $f_{i}$ is the volume fill fraction of the inclusion (metal) in the composite. However, if the metal concentration is high, i.e., c∼a, strong collective dipolar interactions between the nanoparticles take place and the MG theory fails to predict the correct optical response of the nanomaterial.

Another effective medium theory—Bruggeman model treats the host and the inclusion symmetrically [31]. In this model, each particle of either the host or the guest medium is assumed to be embedded in an effective medium with dielectric permittivity $\varepsilon_{\mathrm{eff}}$. The mathematical expression of $\varepsilon_{\mathrm{eff}}$ is given by:

(4) $$f_{1}\frac{\varepsilon_{1}-\varepsilon_{\mathrm{eff}}}{\varepsilon_{1}+2\varepsilon_{\mathrm{eff}}}+f_{2}\frac{\varepsilon_{2}-\varepsilon_{\mathrm{eff}}}{\varepsilon_{2}+2\varepsilon_{\mathrm{eff}}}=0,$$

where $\varepsilon_{1}$ and $\varepsilon_{2}$ are the dielectric permittivities of the constituent materials 1 and 2 respectively, $f_{1}$ and $f_{2}$ are their volume fill fractions. Equation (4) reduces to the Maxwell-Garnett relation i.e., to Equation (3) in the limit $f_{1}\ll f_{2}$. Due to the symmetrical treatment of the host and the inclusion, Bruggeman model can describe the optical response beyond the percolation threshold although it cannot predict plasmon/geometrical resonances.

**Figure 3:**
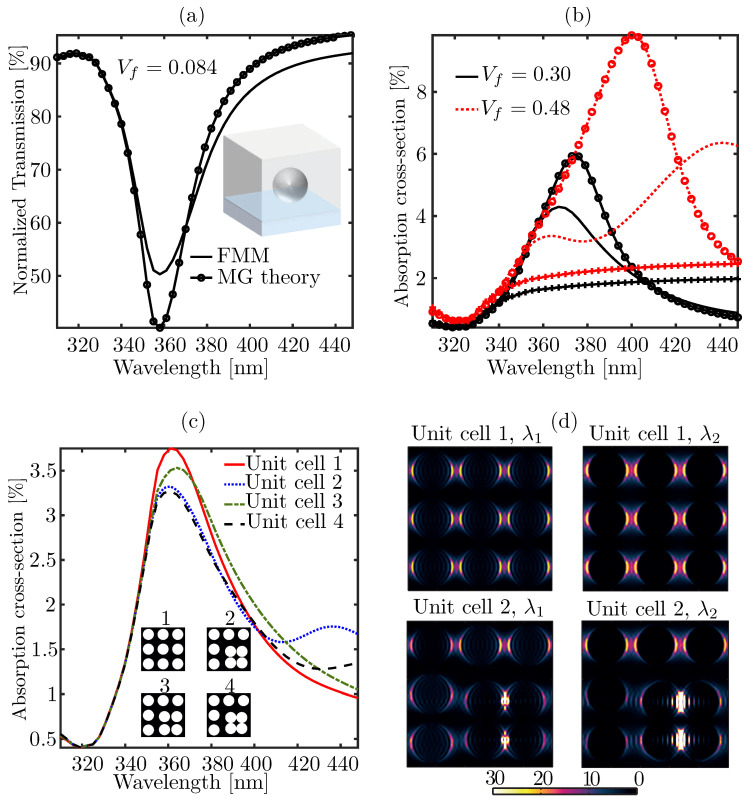
Comparison of the optical properties of a glass-metal nanocomposite (GMN) medium modeled with the effective medium approaches and the rigorous grating theory—(**a**) spectral efficiency in transmission by a GMN with a low metal (silver) fill fraction of 8.4% ($V_{f}$ = 0.084) modeled with MG theory and FMM. The shape of the silver inclusions in the host medium (air) is assumed to be spherical. The inset shows the unit-cell of the periodic structure used for the FMM-based approach. (**b**) Absorption cross-section spectra of a similar GMN monolayer with two different and relatively higher (as compared to that used in (**a**)) metal inclusions of 30% (solid black lines) and 48% (dotted red lines) respectively, modeled with MG theory (marker type—open circles ‘∘’), Brueggemann theory (marker type—diamonds ‘⧫’), and FMM (no marker), (**c**) absorption cross-section spectra of a monolayer GMN with $V_{f}$ = 0.3 calculated with FMM for four different unit-cell configurations (cross-sections through the equators of the nanospheres) as shown in the inset. Each unit-cell contains nine silver nanospheres. The unit-cell configuration 1 is identical to the example included in (**b**). Specifically, the solid red curve in (**c**) is identical to the solid black curve in (**b**), (**d**) *E*-field energy density distributions across the cross-sectional planes through the equators of the silver spheres for unit-cell configuration 1 at $\lambda_{1}$ = 363 nm (upper row left) and $\lambda_{2}$ = 435 nm (upper row right). Similar plots for unit-cell configuration 2 at $\lambda_{1}$ (lower row left) and at $\lambda_{2}$ (lower row right).

We start to investigate with a periodic monolayer of silver spheres of radius a=24 nm placed on top of a glass substrate of refractive index $n_{s}=1.52$ and surrounded by air (n=1). The refractive index data of Ag is taken from ref. [47]. The volume fill fraction $V_{f}$ of silver particles can be varied by changing the inter-particle separation *c*. Let us first assume that c=D=120 nm, i.e., the size of the unit cell in the inset of Figure 3a is 120 nm×120 nm and it contains only one silver particle. Figure 3a shows the normalized transmission spectra for *y*-polarized light under normal incidence calculated using FMM (black line without any marker) and the MG theory (black line with open circles). We notice that for small $V_{f}$ ($V_{f}=0.084$), these two results are in close agreement and the MG theory can accurately estimate the spectral peak position of the plasmon resonance. This particular structure was analyzed in ref. [33] using the Finite-Difference Time-Domain method (FDTD), and our FMM results agree very well with the FDTD calculations—Figure 2a in ref. [33]. We now change $V_{f}$ by changing the inter-particle distance (*D*). Figure 3b shows the resulting absorption cross-section spectra, defined as $\sigma_{\mathrm{abs}}=-\left(1/a\right)\log\left(\eta_{t0}\right)$, where $\eta_{t0}$ is the normalized transmittance in 0-th order. To make the plots in Figure 3b we used FMM (solid black and red dotted lines without any marker) and two different effective medium theories—MG theory (lines with open circles) and Bruggeman model (lines with filled diamond-shaped markers). Clearly, the mismatches between the results obtained with FMM and the effective medium approaches (MG theory and Bruggeman model) increase with increasing $V_{f}$. In particular, the FMM result at $V_{f}=0.48$ shows broadening of the absorption spectrum, which can be attributed to collective plasmon oscillations in the array of silver particles. Such types of spectral features cannot be predicted either by the MG theory or the Bruggeman model.

Next, we study the effects arising from the clustering of the Ag particles, i.e., behavior close to the percolation threshold, on the absorption spectra. For this, we consider a unit cell of size D=190 nm containing nine silver nanoparticles with four distinct arrangements as illustrated in the inset of Figure 3c, where the unit-cell configurations 1–4 show the cross-sections across the mid xy planes (equatorial planes) of the particles. The volume fill fractions of Ag inclusions is taken as $V_{f}=0.3$ in all cases, i.e., we only change the positions of the nanoparticles inside the unit cells. For the arrangement marked with ‘1’, all particles are equally spaced. This particular configuration was already studied in Figure 3b. Figure 3c shows the absorption cross-section spectra for all four configurations. For config. 2, the gaps between the boundaries of the four Ag particles in the lower right corner of the unit cell are small (1.4 nm), so that this cluster is close to the percolation threshold. As a result, collective plasmon oscillations take place, which are manifested in the additional absorption peak around λ=435 nm (blue dotted line) and strong electric field confinement in the gap. For config. 3, the gap size is increased to 5.2 nm, and we notice that the spectral manifestation of the collective plasmon resonance vanishes (green dash-dot line). This occurs also in config. 4 (black dashed line), where the clustered particles just touch each other in both *x* and *y* directions. To investigate the outset of collective plasmon oscillations further, we plot in Figure 3d the localized electric energy density distributions across the mid-planes of the monolayer composite configurations 1 and 2 at two different wavelengths $\lambda_{1}=363$ nm and $\lambda_{2}=435$ nm. Figure 3d for config. 2 shows higher local electric field concentrations in the small gaps between the clustered particles, which are due to the collective plasmon resonances. At $\lambda_{2}=435$ nm, a stronger field concentration in the gap is observed, which gives rise to an additional absorption peak (weaker as compared to the first absorption peak at $\lambda_{1}$) around $\lambda_{2}=435$ nm as can be seen from Figure 3c.

### 3.3. Ag Nanorods Embedded in a Kerr Nonlinear Host Matrix

**Figure 4:**
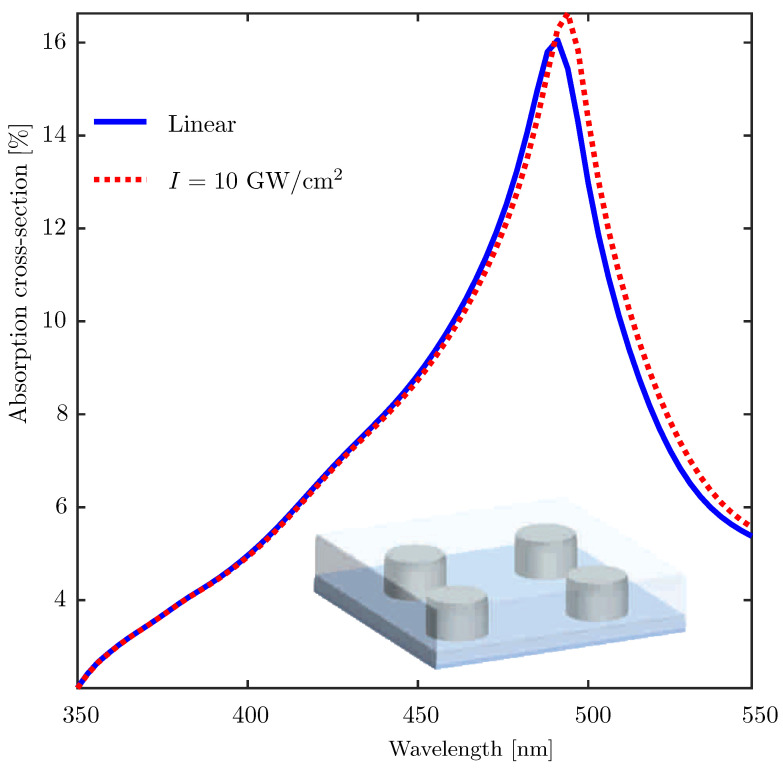
Power-dependent absorption cross-section spectra of a monolayer composite medium consisting of silver nanocylinders embedded in a polymer host medium calculated using the FMM-based approach. The host medium is assumed to possess only Kerr-type optical nonlinearity. The absorption spectra for the incident field intensity I=10 GW/sq. cm, i.e., the dotted red curve shows enhancement and red-shift of the absorption spectrum as compared to the linear optical case.

To demonstrate the performance of our approach for studying the nonlinear optical properties of nanocomposites, as a final example, we consider a regularly arranged monolayer of silver nanorods embedded in a nonlinear polymer medium which possesses Kerr-type optical nonlinearity. The structure geometry is shown in the inset of Figure 4. The linear refractive index and the third-order susceptibility of the host matrix are assumed to be $n_{d}=1.7$ and $\chi^{3}=10^{-17}m^{2}/V^{2}$, respectively. The radius and height of the silver nanorods are a=26 nm and h=50 nm, and the separation between the centers of the neighboring nanorods is d=65 nm both along *x* and *y* directions. The nanorods extend throughout the nonlinear medium, i.e., the thickness of the modulated region (region II in Figure 1) is also 50 nm. The substrate medium is glass with refractive index $n_{s}=1.47$. Figure 4 shows the absorption cross-section spectra of the composite layer. The two plots correspond to the linear case (blue solid line) and a nonlinear case with the intensity of the incoming plane wave taken to be $I=10\mathrm{GW}/{\mathrm{cm}}^{2}$ (red dotted line). Figure 4 shows that the plasmon-enhanced optical Kerr effect manifests itself in the enhancement and red shift of the absorption peak due to an increase of the effective refractive index of the polymer material. In this example, Kerr nonlinerity of only the host material was taken into account. However, it is also possible to model either the inclusion particles or both the inclusion and the host as Kerr nonlinear media, as long as the constituent materials are isotropic/amorphous [43].

## 4. Summary

In summary, we have developed a technique to accurately estimate the optical properties of nanocomposite materials using the Fourier Modal Method, which is a well known numerical tool for rigorous modeling of diffractive optical structures. Arbitrary particle geometries and their random arrangements, effects of particle clustering, and geometric/plasmonic resonances can be modeled seamlessly using the proposed approach. Furthermore, this method can be used to engineer large effective optical nonlinearities at the nanoscale, which is crucial for low power nonlinear photonic devices. In the examples provided herein, we have demonstrated the power of our approach by the considered monolayered nanocomposites containing dielectric and metallic constituents, and also nonlinear optical materials. This method can be readily extended (at the cost of increased computation time) to nanocomposites with arbitrary three-dimensional arrangements of inclusions with the introduction of a longitudinal (along the propagation direction) unit cell. However, the proposed approach can be combined with machine learning-based optimization algorithms for the inverse design of nanocomposites at reduced computation time, although with higher accuracy. The developed tool can also be used for the design and analysis of periodic structures with deterministic randomness and large area supercell metasurfaces. Such types of structures offer an almost unexplored research area for the manipulation and detection of localized electromagnetic fields and light scattering phenomena on planar photonic chips with potential applications in space technologies, imaging, microscopy, and augmented reality/vitual reality (AR/VR) industries.

## Figures and Tables

**Figure A1:**
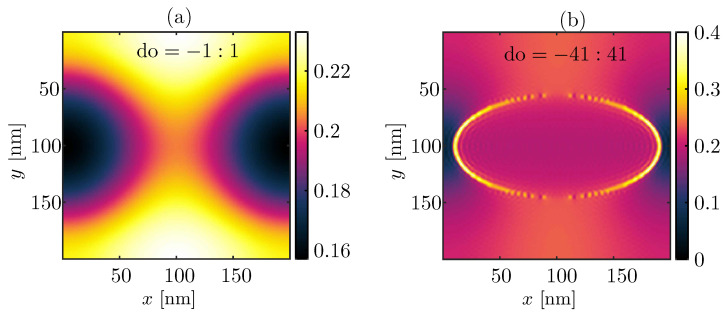
Electric energy density maps immediately behind the test mesoporous Si structure with (**a**) do=−1:1 and (**b**) do=−41:41.

**Figure A2:**
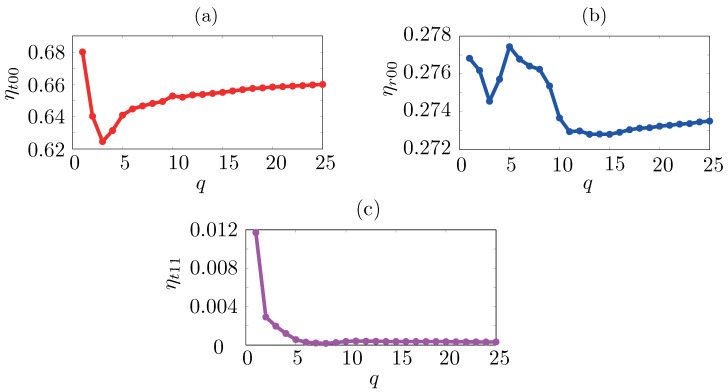
Diffraction efficiencies (**a**) $\eta_{t00}$, (**b**) $\eta_{r00}$, and (**c**) $\eta_{t11}$ plotted with respect to the number of Fourier coefficients used in the FMM calculations. The test object is a monolayer mesoporous silicon on a silicon substrate.

**Figure A3:**
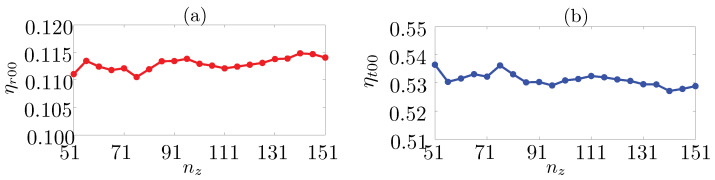
Diffraction efficiencies (**a**) $\eta_{r00}$ of the zeroth reflected order and (**b**) $\eta_{t00}$ of the zeroth transmitted order plotted against the number of layers $n_{z}$ in the FMM analysis.

## Data Availability

Data is available on request. Please contact the corresponding author.

## Supplementary Materials

The following are available online at https://www.mdpi.com/article/10.3390/ma14216359/s1. Video S1: Numerical convergence of electric energy density in the near field against the number of diffraction orders retained in FMM calculations.

## Appendix A. Convergence Tests

### Appendix A.1. Near Field Intensity vs. Number of Fourier Coefficients: Por-Si

To check the numerical convergence of the electric field intensity at the exit plane of the nanocomposites (near *E*-field) against the number of Fourier coefficients (*p* and *q* in Equations (1) and (2)) retained in the FMM calculations, as a test object we took a mesoporous silicon layer containing cylindrical air pores. The pores are assumed to be arranged in a perfectly periodic lattice and their cross-sections (in xy pane) are assumed to be elliptical. The major and the minor axes of the ellipses are assumed to be oriented along the Cartesian *y*- and *x*-axes with their lengths 90.3 nm and 180.6 nm respectively. The size of the unit cell is 200 nm×200 nm, and the thickness of the porous layer is 300 nm. We assume that a *y*-polarized plane wave with λ=1550 nm is normally incident on the porous layer from air. The substrate material is silicon with refractive index n=3.5. The transmitted electric field intensity distribution across the unit cell is investigated right after the porous layer i.e., we study the near *E*-field distribution in the substrate material. The numerical convergence test results are presented in form of an .AVI video file (Supplementary Materials). The text ‘do’ in the video denotes the number of Fourier coefficients retained in the FMM simulations. Same number of Fourier coefficients are used for *y* and *x* directions, which is 2q+1 in each direction when we retain orders −q,…,q, where $q=\max\left(\mathrm{do}\right)$ i.e., *q* is the highest order Fourier coefficient used in the calculations. Figure A1a,b show the starting and the end frames of the attached AVI video file respectively, which illustrate the normalized (to the incident energy density) electric energy density maps immediately behind the porous layer for q=1 (starting frame), and q=41 (end frame). Clearly from the video, we need at least 2q+1∼63 Fourier coefficients (do=−31:31) along both *y* and *x* direction to ensure satisfactory numerical convergence of the optical near field in this specific case.

### Appendix A.2. Diffraction Efficiencies vs. Number of Fourier Coefficients in FMM: Por-Si

In Figure A2, we plot the efficiencies of some selected diffraction orders as a function of *q* retained in the FMM calculations. The test object is a mesoporous silicon monolayer with perfectly periodic arrangement of the pores: the size of the unit cell is 2000 nm×2000 nm, the unit cell contains only one air void, the layer thickness is 300 nm, and *y*-polarized plane wave with λ=1550 nm is assumed to be normally incident from air. In plots Figure A2a,c, we consider $\eta_{t00}$, $\eta_{r00}$, and $\eta_{t11}$, which are the diffraction efficiencies of 0-th transmitted order, 0-th reflected order, and one of the first (11) transmitted orders, respectively. It is clear from the plots that q≈20, i.e., ∼41 Fourier coefficients along *x* and *y* directions ensure numerical convergence of $\eta_{t00}$ and $\eta_{r00}$, while q=10 already gives satisfactory convergence for $\eta_{t11}$ (note the horizontal scales).

### Appendix A.3. Diffraction Efficiencies vs. Number of Layers: Ag Spheres on Glass

Next, we study numerical convergence of far-field diffraction efficiencies as a function of the number of layers ($n_{z}$) along the propagation direction (here *z*) used in the FMM calculations (Figure A3). As a test object, we take the structure used to plot the curve with $V_{f}=0.48$ in Figure 3b of the main text, i.e., a periodic monolayer of silver spheres of radius a=24 nm placed on top of a glass substrate of refractive index $n_{s}=1.52$. The size of the unit cell containing only one Ag particle is 50 nm×50 nm, and the layer thickness is 48 nm. To ensure numerical convergence in the transverse direction, we use 2q+1=41 Fourier coefficients and a dense sampling in xy plane with sub-cell size of 0.15 nm×0.15 nm. The diffraction efficiencies of both reflected and transmitted zeroth orders converge for $n_{z}\approx 100$ (note the horizontal scales).

## Appendix B. Computing Time and Resources

Numerical simulations were performed using in-house developed computer programs in MATLAB, run over a PC with two Intel Xeon X5690 processors each with 6 cores, and 192 GB RAM. Here we provide an estimation of run time. Structural symmetries can be used to reduce the computational workload in FMM calculations as they allow reformulation of the eigenvalue problem into a more compact form [42].

- With do=−10:10 along *x* and *y*, $n_{z}=151$, and transverse sampling $n_{x}=n_{y}=513$, the field calculation across the mid-plane of Ag nanospheres at each wavelength took about 792 s without implementing symmetry conditions and about 169.8 s if $C_{2v}$-type symmetry conditions are used as implemented in ref. [42].
- With do=−20:20 and other parameters same as above, the calculation took about 24,787 s per wavelength without symmetry conditions and about 1195 s with the symmetry conditions.

However, we emphasize that the computation time might be somewhat different in different runs and depend also on the actual number of programs being run simultaneously.
